# Improved adherence with Medicines Use Review service in Slovenia: a randomized controlled trial

**DOI:** 10.1186/s12913-021-06223-8

**Published:** 2021-03-22

**Authors:** Urška Nabergoj Makovec, Igor Locatelli, Mitja Kos

**Affiliations:** Department of Social Pharmacy, University of Ljubljana, Faculty of Pharmacy, Askerceva cesta 7, 1000 Ljubljana, Slovenia

**Keywords:** Medication review, Medicines Use Review, Adherence, Drug related problems, Community pharmacy

## Abstract

**Background:**

Based on several existing patient-oriented activities, Medicines Use Review (MUR) service was standardized and officially adopted in Slovenia in 2015. Service aims to provide adherence support and ensure safe and effective medicines use. Therefore, the aim of the study was to evaluate the benefits of MUR in Slovenia, primarily the impact on medication adherence.

**Methods:**

A randomised controlled trial was performed in community pharmacies to compare MUR with standard care. Patients were randomised into either the test (patients received MUR by a certified MUR provider at visit 1), or control group. The study primary outcome was self-reported adherence to multiple medications, assessed by electronic ©Morisky Widget MMAS-8 Software at the first visit (V1) and after 12 weeks (V2). A sub-analysis of intentional and unintentional non-adherence was performed. MUR impact was defined as the relative difference in ©MMAS-8 score after 12 weeks between the test and control group. A multiple linear regression model was used to predict MUR impact based on baseline adherence (low versus medium and high). Several secondary outcomes (e.g. evaluation of drug-related problems (DRPs)) were also assessed.

**Results:**

Data from 153 (V1) and 140 (V2) patients were analysed. Baseline adherence was low, moderate and high in 17.6, 48.4 and 34.0% patients, respectively. In the low adherence subpopulation, test group patients showed a 1.20 point (95% CI = 0.16–2.25) increase in total ©MMAS-8 score (*p* = 0.025) compared to control group patients. A 0.84 point (95% CI = 0.05–1.63) increase was due to intentional non-adherence (*p* = 0.038), and a 0.36 point (95% CI = − 0.23-0.95) was due to unintentional non-adherence (*p* = 0.226). Additionally, statistically significant decrease in the proportion of patients with manifested DRPs (*p* < 0.001) and concerns regarding chronic medicines use (*p* = 0.029) were revealed.

**Conclusion:**

MUR service in Slovenia improves low medication adherence and is effective in addressing DRPs and concerns regarding chronic medicines use.

**Trial registration:**

ClinicalTrials.gov - NCT04417400; 4th June 2020; retrospectively registered.

**Supplementary Information:**

The online version contains supplementary material available at 10.1186/s12913-021-06223-8.

## Background

Medication review (MR) services are recognized cognitive pharmaceutical services that aim to optimize medicines use and improve health outcomes [1, 2]. Pharmaceutical Care Network Europe (PCNE) defined different types of MR, among them intermediate MR (type 2), commonly known as medicines use review. It involves a structural assessment of a patient’s medicines, based on medication history and information provided by the patient or their carer, and with a specific focus on medication adherence and proper medicines use [3].

Poor medication adherence (‘non-adherence’) is a proven contributor to poor health outcomes [4]. Non-adherence is a complex behaviour that can be grouped into two sub-behaviours - intentional and unintentional non-adherence [5–8]. Factors influencing both behaviour groups can be categorized as either external (e.g. therapy characteristics, health system) or internal (e.g. patient attitudes and beliefs regarding medicines) [4, 7]. In order to improve patient adherence, a multilevel approach should be taken that identifies specific individual factors for each patient and customizes interventions to challenges that are recognized for that patient [5–7].

Literature shows, medicines use review services can improve adherence [9–17], since factors that influence safe and effective medicines use are discussed between pharmacist and patient, and tailored interventions to resolve identified drug related problems (DRPs) are performed [16–18]. According to the PRACTISE study [1, 2], medicines use review services are offered in 14 European countries, among them in Slovenia. Almost 300 Slovenian community pharmacists throughout the country (data from 2020) are certified to provide a standardized Medicines Use Review (MUR) service; however, provision rates vary and service is not remunerated [19, 20]. Implementation of MUR service within Slovenian pharmacy practice has been evaluated in several studies [19, 21], among which a randomized controlled trial (RCT) was designed with the aim to evaluate the benefits of MUR service in Slovenia, with a specific focus on evaluating the impact of MUR on patient adherence to medicines.

## Methods

### Study design and setting

A prospective, non-blinded RCT with a parallel group design was performed to compare MUR to standard care. The study took place in community pharmacies across Slovenia, that provide MUR service according to the standard operating procedure for MUR (SOP MUR) [20]. Study was performed by three partners (i) community pharmacists, certified to provide MUR (**‘study pharmacists’**), who recruited patients and provided MUR, (ii) final year pharmacy students (**‘study interviewers’**), who interviewed patients to collect data and administer questionnaires and (iii) researchers from the University of Ljubljana, Faculty of Pharmacy (**‘study coordinators’**), who designed and run study protocol and performed data analysis and study report following CONSORT statement guidelines [22]. All study pharmacists and study interviewers received full training prior and within the study.

**Fig. 1:**
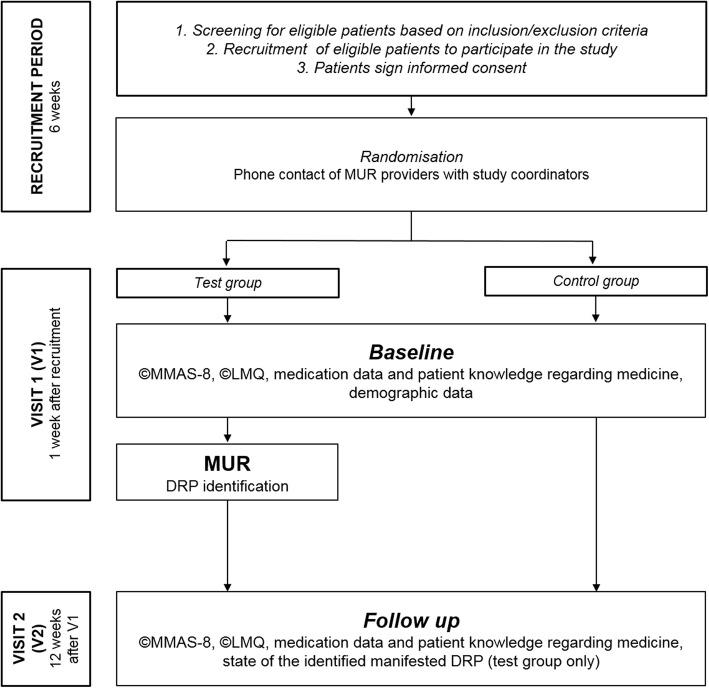
The RCT timeline

Enrolled patients attended two visits: (i) visit 1 (V1), when baseline data was collected and patients randomized into test group received MUR as per SOP MUR; (ii) visit 2 (V2) after 12 weeks as a follow up visit. After completion of the RCT, control group patients received MUR in order to comply with ethical standards of healthcare. A detailed timeline of the study elements is presented in Fig. 1.

### Eligibility criteria

Patients were eligible for study inclusion if they: (i) had been taking at least one prescription medicine for a chronic condition for at least six months, (ii) were deemed to be suitable for MUR by a study pharmacist at regular dispensing of medicines or random visit to the pharmacy; (iii) were able to communicate in Slovenian; and (iv) were at least 18 years old. The inclusion of patients was performed in accordance with the SOP MUR [20], where examples of patients, who would benefit from the service are presented and pharmacist are trained to recognized them (Additional file 1: Appendix A). This approach to enrolment was used to reflect how patients are generally offered MUR service in Slovenia.

Patients were excluded if: (i) they needed MUR sufficiently urgently that service delays due to randomization would risk their health, (ii) referral for MUR by a healthcare professional (general practitioner, nurse, etc.), (iii) the patient’s carer, rather than the patient themselves, was able to attend the interviews and receive MUR, (iv) they have previously received MUR or an advanced medication review, (v) they had difficulties understanding, communicating or other issues (vision, hearing, swallowing) that might affect study outcomes.

Randomization was performed centrally by the study coordinators. When a patient was enrolled, the study pharmacist contacted study coordinator via telephone and the coordinator revealed allocation of the patient. Each pharmacist was considered as one study centre to ensure even distribution of MURs across them. Two randomly generated blocks of four randomization allocations were assigned to each pharmacist prior to the study. A block of four was compounded of two control and two MUR allocations in different variations (e.g. test-control-test-control) [23]. When a pharmacist run out of allocations, new blocks were generated. If a study patient withdrew prior to V1, the assigned allocation was excluded. Prior to data analysis, the study and control group were tested for potential baseline differences in patient characteristics.

### Intervention

The MUR service (defined in the SOP MUR [20]) is classified as a type 2a review (PCNE typology [3]) and it is performed based on medication history and information provided by the patient or their carer in a purposely scheduled and private conversation with the pharmacist. During the MUR interview, the pharmacist completes a working sheet that records information regarding medicines, identified DRPs and recommended interventions. After the MUR interview, patients are provided with a personal medicines card that contains all the information that is needed to support effective and safe medicines use (dosing regimen, taking with/without food, special warnings, recommendations, etc.) [19, 20]. Pharmacists, who intend to provide MUR, must first complete a specific educational program to ensure their competency for service provision.

In the study, ‘standard care’ was defined as pharmacist advice and instructions on medicines use at the dispensing according to the Slovenian Pharmacy Practice Act [24].

### Primary outcome measure

The primary outcome of this study was **self-reported medication adherence** to multiple medications, evaluated using ©Morisky Widget MMAS-8 Software [25–29] at two timepoints: baseline (V1) and after 12 weeks (V2). The questionnaire was adapted for generic use, across all prescription medications for regular use. Patients scores were classified as follows: < 6 low adherence, 6 to < 8 moderate adherence, and = 8 high adherence. A licence agreement to use ©Morisky Widget MMAS-8 Software was obtained prior to the study.

The effect of MUR on total ©MMAS-8 score, intentional non-adherence ©MMAS-8 score and unintentional non-adherence ©MMAS-8 score was defined as the mean relative difference in each score after 12 weeks (V2-V1) between the test and control group. The differences in adherence scores were first tested with the Mann-Whitney U test (α = 0.05), before using a multiple linear regression model to predict the effect of MUR on adherence scores based on baseline adherence (low versus moderate and high baseline adherence). The linear regression model included an interaction variable between patient allocation (control versus test group) and baseline adherence level as a dichotomous variable (low versus moderate and high). By including the interaction variable, the model was able to assess how the effect of patient allocation on adherence is affected due to the baseline adherence level, and vice versa [30]. Furthermore, a multiple linear regression model was used to evaluate how patient characteristics are related to baseline adherence level. All statistical tests and regression models were performed using IBM SPSS v25 [31].

### Secondary outcome measures

#### Drug related problems

A before-after analysis was performed as part of RCT to asses DRPs identified in test group. Patients DRPs were identified during MUR (V1) and classified based on pharmacists’ descriptions on MUR documentation with the use of Slovenian classification DRP-SLO-V1 [32]. At follow-up (V2), test group patients were interviewed about the current state of their identified manifested DRP (mDRP). The proportion of mDRP, the proportion of patients with at least one mDRP, and the proportion of patients with a change in mDRP risk level were calculated for both visits and compared using a one sample binomial test (α = 0.05). The analysis made two assumptions: (i) no new DRPs (manifested or potential) arose between the visits, and (ii) DRPs of dropout patients and DRPs with unknown outcomes were excluded. The mDRP risk level was defined as the number of identified mDRP for every patient or the number of persisting mDRP for V1 and V2, respectively. The value of partially resolved mDRP at V2 was 0.5.

#### Other secondary outcomes

The burden related to daily medicines use was assessed using The Living with Medicines Questionnaire version 3 (©LMQ v3) [33], which has been previously translated and pilot tested for use in Slovenian practice [34]. Permission to use LMQ v3 was obtained from its authors. MUR impact on medicine-associated burden was defined as the mean relative difference in ©LMQ, VAS and each domain score between test and control group after 12 weeks (V1-V2). All statistical tests were performed using IBM SPSS v25 [31].

Furthermore, patients were asked three questions regarding purpose of use, daily dose and special warnings for each of their regularly used prescription medicines. Accuracy of answers were checked with either physician instructions on the e-prescription form or Summary of Product Characteristics and presented as proportions of correct and incorrect answers per patient-medicine and proportions of improvement or deterioration in given information between visits for the test and control group. No further characterization or validation of information provided by the patients (e.g. confirming diagnosis with the physician or medical records) were performed.

### Sample size

Sample size was calculated on the basis of a 0.5 point mean difference in ©MMAS-8 score between the test and control group, a one point difference in ©MMAS-8 score for standard deviation, a type 1 error of 5%, and 80% statistical power. This resulted in an estimated sample size of 64 patients in each group. Assuming a 25% dropout rate, we aimed to recruit a total of 170 patients (85 per group) in order to detect improvement in primary study outcome.

### Handling of missing data

Only data from the patients, who attended both visits (V1 and V2), was used in the analysis. Patients were considered as dropout if they were randomized and have not attended V1 or V2. Reasons for dropout were documented, when available. Furthermore, patients were excluded from the analysis when a breach of protocol was noted. Single missing answers in the ©MMAS-8 or ©LMQ were imputed with the overall median value for the given question or statement. Cases of imputation were documented and reported [35].

### Ethics approval and consent to participate

The study was approved on 16th June 2016 by the Republic of Slovenia National Medical Ethics Committee (NMEC) (registry number: MZ 0120–321/2016–2, KME 49/06/16). Written informed consent was obtained from all individual participants included in the study upon explanation of the aim and nature of the study.

## Results

The study ran from May to September 2017. At the time of the study around 100 pharmacists were certified to provide MUR and all received invitation to participate in the study. Thirty-one pharmacists from 30 different community pharmacies across Slovenia responded. The study also involved 18 study interviewers.

### Study population characteristics

**Fig. 2:**
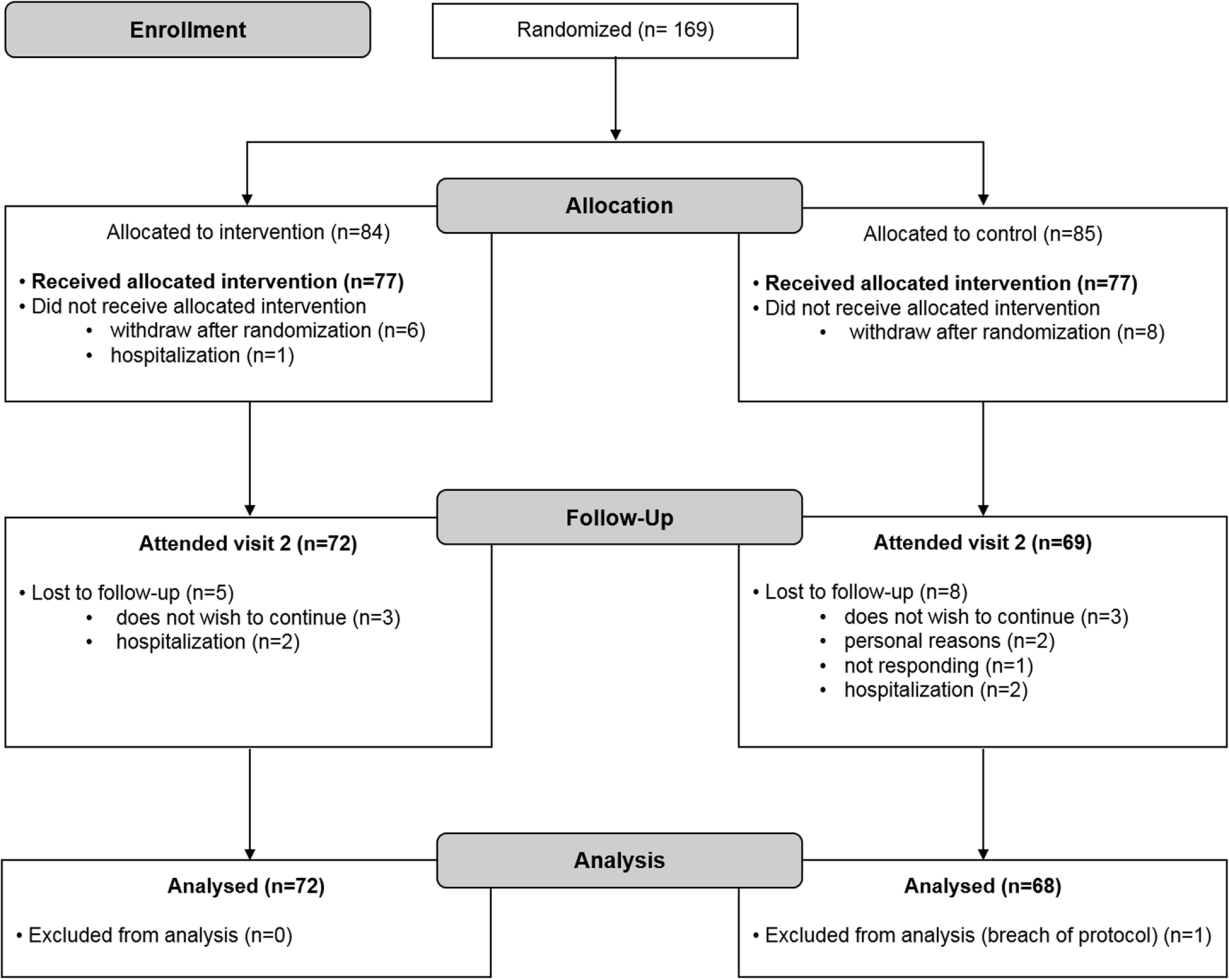
CONSORT flow diagram detailing patient flow through the study, including reasons for withdrawal

A total of 140 study patients were included in the analysis. Total dropout rate was 17% – 8.9% between randomization and V1 and 8.4% between V1 and V2. Data from one control group patient was excluded in the analysis phase since that patient received MUR at V1 due to signs of serious health issues (Fig. 2).

Polypharmacotherapy was the main reason (single or in combination) for patient inclusion in the study (74%), followed by patient need for additional information (30%). Adherence and adverse drug reactions were given as reasons in 7% and 10% of patients, respectively. The medicines taken by included patients for chronic therapy were in 48.9% for cardiovascular system, followed by medicines for alimentary tract and metabolism (16.5%), blood and blood forming agents (11.5%) and nervous system (8.4%). Other medicines groups were represented in less than 4% each. No statistically significant differences in patient baseline characteristics between the groups were found (Table 1). Details of the missing data handling are presented in the Additional file 1: Appendix B*.*

**Table 1:** Baseline characteristics of the study population grouped into control and test group, at V1 (*N* = 153) and an inter-group comparison

Baseline characteristics	Control group (***N*** = 76)		Test group (***N*** = 77)		***p*** value
Age [years; mean (SD) and range]	67.8 (9.56)	41–89	68.7 (10.3)	39–86	0.479
Gender [female; n/%]	38	50.0	49	63.6	0.089
Employment status [retired^a^; n/%]	63	82.9	67	87.0	0.356
Education [n/%]					
*Elementary school*	14	18.4	13	16.9	0.775
*High school*	36	47.4	41	53.2	
*College*	15	19.7	13	16.9	
*University*	10	13.2	9	11.7	
*Do not want to answer*	1	1.3	1	1.3	
Number of Rx medicines for regular use [median and range]	7	2–13	7	2–13	0.644
Number of units of medicines per day [median and range]	8	2–23	8	2–21	0.970
How many times per day patients take their medicines [n/%]					
*Once per day*	1	1.0	2	3.0	0.970
*Twice per day*	46	61.0	41	53.0	
*Three times per day*	27	36.0	32	42.0	
*Four times per day*	2	3.0	2	3.0	
Different pharmaceutical forms [n/%]					
*Different pharmaceutical forms*	33	43.0	41	53.0	0.224
*Only tablets and capsules*	43	57.0	36	47.0	
(Co)paying for medicines [yes; n/%]	47	61.8	37	48.1	0.087
Help with medicines use [yes; n/%]	7	9.2	8	10.4	0.806
Self-reported current health status [n/%]					
*Very poor*	0	0.0	3	3.9	0.999
*Poor*	5	6.6	5	6.5	
*Medium*	47	61.8	43	55.8	
*Good*	20	26.3	23	29.9	
*Very good*	2	2.6	2	2.6	
*Don’t want to answer*	2	2.6	1	1.3	

^a^one patient in test group (1.13%) did not wish to answer

### Primary outcome: self-reported adherence according to ©MMAS-8

Low, moderate and high levels of adherence were demonstrated at V1 for 17.6, 48.4 and 34.0% of study patients, respectively. At follow up (V2) 17.1, 36.4 and 46.4% patients had low, moderate and high adherence, respectively. ©MMAS-8 score results, presented per study group, are shown in Table 2. Patients with higher educational levels (*p* = 0.032) and with better self-reported health status (*p* = 0.044) showed higher baseline adherence levels (Additional file 1: Appendix C). The Mann-Whitney U test showed that there were no statistically significant differences between the groups in total ©MMAS-8 score, intentional non-adherence and unintentional non-adherence (*p* = 0.941, *p* = 0.872 and *p* = 0.813, respectively).

**Table 2:** Total ©MMAS-8 score, intentional non-adherence ©MMAS-8 score and unintentional non-adherence ©MMAS-8 score at V1(N = 153) and V2 (N = 140)

©MMAS-8 score	Visit 1				Visit 2			
	*Test group (N = 77)*		*Control group (N = 76)*		*Test group (N = 72)*	*Control group (N = 69)*		
**Total** *[mean (SD) and range]*	6.96 (1.07)	3.25–8	6.86 (1.21)	2.75–8	7.12 (1.18)	2.75–8	6.91 (1.29)	2.75–8
**Intentional** *[mean (SD) and range]*	3.48 (0.79)	1–4	3.49 (0.86)	1–4	3.56 (0.8)	0–4	3.47 (0.84)	0–4
**Unintentional** *[mean (SD) and range]*	3.48 (0.65)	1.75–4	3.37 (0.68)	1.75–4	3.56 (0.66)	1.75–4	3.44 (0.73)	1.75–4

#### Difference between the visits (V2-V1) based on baseline level of adherence

Twenty-five patients (test = 10; control = 15) with low baseline adherence and 115 patients (test = 62; control = 53) with moderate and high baseline adherence attended both visits (Additional file 1: Appendix D). A multiple regression model showed that test group patients with low baseline adherence improved their overall adherence by 1.20 points between V1 and V2 when compared to control group patients (*p* = 0.025). A 0.84 point increase was due to intentional non-adherence (*p* = 0.038), and a 0.36 point due to unintentional non-adherence (*p* = 0.226) (Table 3).

**Table 3:** Multiple linear regression models to predict difference in adherence between visits (V2-V1) for total ©MMAS-8 score, intentional non-adherence ©MMAS-8 score and unintentional non-adherence ©MMAS-8 score (*N* = 138)

	Total			Intentional			Unintentional		
	*B*	*95% CI for B*	*p value*	*B*	*95% CI for B*	*p value*	*B*	*95% CI for B*	*p value*
*Constant*	−1.059	−3.05 – 0.93	0.294	−0.335	−1.84 – 1.17	0.661	−0.724	−1.84 - 0.39	0.201
***MUR effect for low adherent patients***	1.201	0.16–2.25	**0.025**	0.841	0.05–1.63	**0.038**	0.360	−0.23 - 0.95	0.226
***Baseline adherence***^*a*^	−0.973	−1.68 - -0.26	**0.008**	−0.540	−1.08 - -0.00	**0.049**	−0.433	−0.83 - -0.04	**0.033**
***Interaction*** [MUR effect* baseline adherence]	−1.229	−2.37 - -0.09	**0.035**	−0.812	−1.68 – 0.05	0.066	−0.416	−1.06 - 0.22	0.200
***Self-reported current health status***^*b*^ [1–5]	0.342	0.03–0.65	**0.032**	0.290	0.05–0.53	**0.016**	0.052	−0.12 - 0.23	0.558
***Gender*** *(male* vs. *female)*	0.063	−0.39 – 0.52	0.785	−0.065	−0.41 – 0.28	0.712	0.128	−0.13 - 0.38	0.325
***Age***^*b*^ *[years]*	0.026	−0.002 – 0.05	0.068	0.006	−0.02 – 0.03	0.587	0.020	0.00–0.04	**0.012**
***Education***^*b*^ [1–4]	−0.194	− 0.44 – 0.05	0.114	0.007	−0.18 – 0.19	0.936	−0.201	− 0.34 - -0.07	**0.004**
***Employment status*** *(retired* vs. *non-retired)*	−0.403	−1.18 – 0.37	0.305	−0.198	−0.78 – 0.39	0.506	−0.206	− 0.64 - 0.23	0.349
***Number of Rx medicines for regular use***^b^	−0.005	−0.10 – 0.09	0.918	0.007	−0.07 – 0.08	0.844	−0.012	−0.07 - 0.04	0.653
***Different pharmaceutical forms***^c^	0.243	−0.20 – 0.69	0.280	0.065	−0.27 – 0.40	0.704	0.179	−0.07 - 0.43	0.157
***(Co)paying for medicines*** *(no* vs. *yes)*	−0.266	−0.68 – 0.15	0.202	−0.391	− 0.70 - -0.08	**0.014**	0.125	−0.11 - 0.36	0.284
*Model summary*	*N = 138; R*^*2*^ *= 0,238; p < 0,001*			*N = 138; R*^*2*^ *= 0,176; p = 0,009*			*N = 138; R2 = 0,216; p < 0,001*		

^a^ baseline moderate and high versus baseline low adherence in control group

^b^ included as scale variable

^c^ different pharmaceutical forms vs. only tablets and capsules

**Fig. 3:**
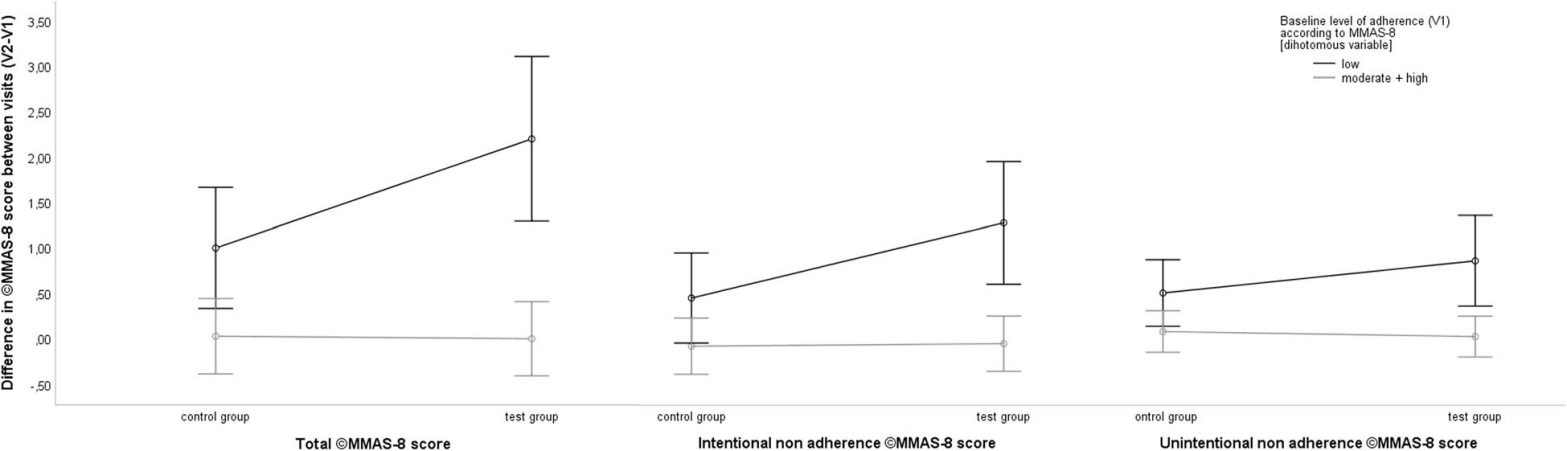
Difference in ©MMAS-8 score (V2-V1), ©MMAS-8 score of intentional non-adherence (V2-V1) and ©MMAS-8 score of unintentional non-adherence (V2-V1). The regression models were adjusted for current health status, gender, age, education level, employment status, number of Rx medicines and co-payment. Error bars indicate 95% CI

The regression models are presented graphically in Fig. 3 and explain the effect of interaction between patient allocation and baseline adherence. The effect of MUR is presented as a slope in ©MMAS difference separately for low and for moderate and high adherent patients. The slope is steeper for low adherent patients (marked effect, difference in total ©MMAS score is 1.2 points) compared to moderate and high adherent patients (no effect, difference in total ©MMAS score is − 0.03 point). The slopes of the intentional and unintentional models should be interpreted equivalently.

### Secondary outcomes

#### Drug related problems

A total of 221 DRPs (130 potential and 90 manifested) were identified in 77 patients from the test group (average 2.87 DRPs per patient). Of these patients, 65% had at least one manifested DRP (mDRP), while 35% only had potential DRPs. The nature of the identified mDRPs, risk factors and performed interventions are presented in detail in Additional file 1: Appendix E*.* At follow up (V2), 53% of the mDRP had been partially or fully resolved, 25% remained unresolved and the outcome was unknown for 22%. The binomial test showed statistically significant difference in the proportion of mDRP (*p* = 0.001), the proportion of patients with at least one mDRP (*p* < 0.001) and the proportion of patients with a change in mDRP risk level (p < 0.001).

#### Other

The overall burden of everyday use of medicines did not decrease as a result of receiving MUR (©LMQ *p* = 0.618; VAS *p* = 0.911). However, patient concerns regarding their daily use of medicines decreased after they received MUR (domain 6 of the ©LMQ), with a statistically significant difference in domain score (V1-V2) between groups (*p* = 0.029) (Additional file 1: Appendix E.).

At the first visit (V1; *N* = 1050 patient-medicines), the purpose of medicine use and daily dose was stated correctly in 90 and 91% of patient-medicines, respectively. Among the correctly stated daily doses, the medication regimen was also correctly stated in 85% of patient-medicines. At second visit (V2; *N* = 939 patient-medicines), understanding of the purpose of their medicines and daily dose improved and deteriorated in equal proportions (5%). Patients stated special warnings regarding their medicines in 15 and 11% of patient-medicines at V1 and V2, respectively. Among the special warnings, adverse event information was stated in 6.5 and 2% of patient-medicines at V1 and V2, respectively (Additional file 1: Appendix E.)

## Discussion

The study and its findings represent an evidence-based approach in implementation of cognitive pharmaceutical service to a pharmacy practice. Several benefits of MUR are demonstrated, including improving patient adherence and reducing DRPs; these findings further strengthen existing evidence of MUR effectiveness [9, 10, 12, 13, 17, 36–38]. Furthermore, it is encouraging that the objectives of MUR services are indeed being achieved, further supporting recognition of the key role of pharmacists in ensuring effective and safe medicines use.

### Benefits and challenges of MUR service

MUR improved adherence in low adherent patients by 1.2 points (©MMAS-8 score) compared to no MUR. Surprisingly, a sub-analysis of intentional and unintentional non-adherence revealed more significant improvements in intentional (0.84 point) than unintentional (0.36 point). The result is additionally supported by the decreased patient concerns regarding long-term medicine use, as established through the ©LMQ domain 6. At the time of writing, the authors were not aware, of any other research of medicine use review services with similar results regarding the adherence sub-behaviours. Several practical approaches (e.g. dosing aids, medication cards) were proven effective in addressing unintentional non-adherence and are usually easier to comprehend and implement by the patients [4, 39, 40]. Therefore, the absence of MUR effect in this sub-type behaviour suggests more effort is needed in the provision of dosing aids, reminder settings and simplifying medication regimens to address factors of unintentional nonadherence. A similar conclusion was drawn by the study of Poly Medication Check service in Switzerland [17].

On the other hand, the intentional non-adherence is usually based on patient beliefs and attitudes towards disease and medicines and is thus generally harder to impact [5, 7, 39]. Even though the nature of pharmacists’ interventions did not require any unusual or innovative approaches, the study pharmacists were successful in addressing patient issues and attitudes, and they were able to provide comfort and adequate support for change in behaviour. The results of this study highlight the value of good communication skills for high quality MUR provision, and pharmacists should, therefore, be encouraged and provided with opportunities to develop and improve their communication skills. For example, it might be beneficial to include training on motivational interviewing in MUR educational program [41, 42].

While this study revealed that MUR has the effect on a subpopulation of low adherent patients, its effect on the total population was not established due to already high baseline adherence - a common characteristic of previous studies into medication review services [9, 17, 18] and adherence interventions [9, 43], which failed to reveal positive effects from interventions. This finding provides strong evidence of the need for more rigorous screening to identify patients with low adherence, and to provide those patients with suitable interventions. A very small proportion of patients received MUR due to non-adherence, indicating insufficient tools and/or training for pharmacists to recognize the signs of non-adherence, or simply that pharmacists are more focused on other aspects of medicines use, such as polypharmacotherapy and the need for additional patient information.

Regular use of a large number of prescribed medicines might be a risk factor for non-adherence [4, 44, 45], although results from this study indicate that it more likely translates into other types of DRPs rather than non-adherence. Large numbers of detected mDRP (40.7%), with two thirds of patients experiencing at least one mDRP, confirm the results of a 2016 MUR implementation study [21] and add to extensive evidence supporting the role of community pharmacists in identifying DRPs [17, 46–49]. It is encouraging that half of them were partially or fully resolved, illustrating the beneficial effects of MUR services and emphasizing the importance of patient follow-up [21]. However, the nature of the identified DRPs, along with the associated interventions, reveals also MUR limitations. The majority of mDRP were adverse events with unclear risk factors (60%), followed by treatment effectiveness issues (18.9%), demonstrating pharmacist limitations such as lack of access to clinical data and the scope of independent interventions to successfully resolve DRPs. For MUR service to take a significant role in patient care, strong collaboration between pharmacists and other healthcare professionals is required.

Patient need for additional medicines information suggests that patients actively search for this kind of information, particularly patients who are aware of the challenges arising from medicines use. Despite this context, MUR providers should actively seek out less proactive patients, who do not typically express a need for medicines information and might be in greater risk for non-adherence and DRPs and will therefore benefit more from MUR. Previous studies investigating patient knowledge about medicines [50–52] demonstrate high levels of patient knowledge regarding basic information (indication, daily dose and medication regimen) and low levels of patient knowledge regarding medicines safety (adverse events, special warnings); results from this study concur with their results. Knowledge as the secondary outcome, lack of validated instruments, high baseline levels of the general medicine information and nonspecific questions regarding safety information, might be some of the reasons that MUR did not affect the outcome in this study. Nonetheless, provision of more detailed medicine safety information is important, and MUR is potentially an excellent tool for pharmacist-patient communication due to the lack of time-pressure, which is a frequent problem during regular dispensing activities when pharmacists tend to focus on providing general information rather than medicine safety information [50].

### Study implementation and population

Since the RCT study design is used infrequently in research of Slovenian pharmacy practice, high dropout rates were expected. Dropout rate was a bit lower (17%) than expected and evenly distributed across the test and control group, as well as other baseline characteristics. The participation of 31 certified MUR providers from 30 community pharmacies enabled coverage of all major regions in Slovenia, strengthening sample representativeness.

The study population represents typical visitor to community pharmacy: retired, elderly people with high-school level education who suffer from multiple chronic diseases that require regular prescription medication. Only a small share experienced adherence issues and higher levels of burden due to daily medicines use, which on one hand, suggest the population, who has generally accepted medicine taking as part of their daily living. On the other hand, demographic and multimorbidity characteristic put them at risk of DRPs, which may lead to further health complications and/or deterioration of their health status. Therefore, the study population provide a good representation of a population that might benefit from receiving MUR.

### Strengths and limitations

This study has several strengths. First, the study takes an RCT design, which is arguably the most powerful design type, allowing intervention comparisons between patients who were randomly allocated to control and test group. Although it was not possible to perform a totally blinded study, a certain degree of blinding was possible during recruitment period thus, there was limited bias in patient recruitment. Secondly, the study had broad inclusion criteria that reflect contemporary, real-life practice of offering MUR service; this, combined with a broad range of MUR service, means the results can be generalized to wider populations. Finally, data was collected by final year pharmacy students who had been recruited as independent study partners; our experiences [53, 54] have shown that patients tend to be more open and truthful towards pharmacy students as they see them as independent, further adding to the credibility and trustworthiness of this study.

On the other hand, the use of another adherence measure might have provided additional insight and further validate our findings [44]. Nonetheless, the ©MMAS-8 is a validated questionnaire and has been reported as one of the most frequently used to asses adherence over multiple medications [55]. Secondly, although a larger patient population would provide additional sample power, a greater impact would only be noticed if a larger number of low adherent patients would have been recruited. However, the aim of the study was to reflect real-life practice and not to focus on specific population. Thirdly, a longer study period would allow us to determine the long-term effect of MUR services on the investigated outcomes, especially how long after MUR the effects start to fade and MUR should be repeated. A retrospective study by Hatah et al. showed that longer duration and several visits predict better adherence scores [36]. Moreover, a systematic review by Huiskes et al. suggest the importance of longer observation and non-cross sectional nature of MR services [56]. Finally, although all possible precautions were taken to reduce study bias it is not possible to exclude the potential ‘observer effect’ [57] on both study patients and study pharmacists as well as that the included patients and pharmacist are generally more proactive regarding their health and work. Furthermore, the same pharmacists, who performed MUR also performed regular medicine dispensing to control group, which could have additionally biased the estimation of MUR effect.

### Implications for pharmacy practice

Based on the study evidence, MUR service should be restructured to increase its effectiveness and efficiency. Firstly, narrower and more specific inclusion criteria would increase service impact. One possibility is to create evidence-based target groups of patients who exhibit challenges in medicines adherence. For instance, previous studies have shown patients with asthma [58, 59] are at risk of low adherence; indeed, Manfrin et al. [10] demonstrated the effectiveness of MUR in the asthma population. Secondly, limited health resources dictate their rational use and cost-effective services. In a recent study, New Medicines Service in UK [16], a complementary MUR service, demonstrated the service to be cost-effective and to have a positive impact on adherence. Taking that example alongside evidence of MUR benefits in Slovenia resulted in a proposal to reorganize MUR services for patients with newly prescribed medicines from specific target groups [60]. Several countries experience challenges related to successful and sustainable implementation of medicines use review and other cognitive pharmaceutical services; the present study results provide additional insights and evidence how to tackle these challenges.

## Conclusion

This study showed that MUR is effective in addressing adherence, especially for low adherence patients. Furthermore, large numbers of mDRP are identified and properly addressed during MUR, and patients feel empowered and less worried about chronic medicines use. The future of MUR services relies in a more rational approach with better recognition of high-need patients, specific evidence-based inclusion criteria and tailored interventions to address issues identified in this study.

## Supplementary Information

**Additional file 1. Appendix A**: Inclusion criteria - examples of patients suitable for the MUR service as per SOP MUR. **Appendix B**: Handling of missing data. **Appendix C**: Multiple linear regression models to predict baseline adherence for total ©MMAS-8 score at V1 (*N* = 129). **Appendix D**: Baseline population characteristics presented per group and per baseline adherence level (low versus moderate and high) for all patients included in the analysis (*N* = 140). **Appendix E**. The detailed results of the secondary outcomes: nature of identified manifested DRPs, risk factors and interventions, ©LMQ and VAS scores and patient information (‘knowledge’) regarding their medicines.

**Additional file 2.** Questionnaires used in the Medicines use Review Service in Slovenia: a randomized controlled trial (RCT MUR SLO).

**Additional file 3.** CONSORT 2010 checklist for RCT MUR SLO.

## Data Availability

The data that support the findings of this study are available from the corresponding author upon request and mutual agreement.

## Abbreviations

DRP: Drug related problem
©LMQ: The Living With Medicines Questionnaire
mDRP: Manifested drug related problem
©MMAS-8: 8-item Morisky Medication Adherence Scale
MR: Medication review
MUR: Medicines Use Review service
PCNE: Pharmaceutical Care Network Europe
RCT: Randomized controlled trial
SOP MUR: Standard operating procedure for Medicines Use Review service in Slovenia
V1: Visit 1
V2: Visit 2
VAS: Visual Analog Scale

## References

[1] Imfeld-Isenegger TL, Soares IB, Nabergoj Makovec U, Horvat N, Kos M, van Mil F, et al. Community pharmacist-led medication review procedures across Europe: characterization, implementation and remuneration. Res Social Adm Pharm. 2019. 10.1016/j.sapharm.2019.11.002. 10.1016/j.sapharm.2019.11.002 31734100

[2] Soares IB, Imfeld-Isenegger TL, Nabergoj Makovec U, Horvat N, Kos M, Arnet I (2020). A survey to assess the availability, implementation rate and remuneration of pharmacist-led cognitive services throughout Europe. Res Social Adm Pharm.

[3] Griese-Mammen N, Hersberger KE, Messerli M, Leikola S, Horvat N, van Mil JWF (2018). PCNE definition of medication review: reaching agreement. Int J Clin Pharm.

[4] Osterberg L, Blaschke T (2005). Adherence to medication. N Engl J Med.

[5] Horne R, Cooper V, Vvileman V, Chan A (2019). Supporting adherence to medicines for long-term conditions a perceptions and practicalities approach based on an extended common-sense model. Eur Psychol.

[6] Horne R, Petrie KJ (2014). Patients' Perceptions of Illness and Treatment: Targets for Interventions to Support Medication Adherence. Int J Behav Med.

[7] Lehane E, McCarthy G (2007). Intentional and unintentional medication non-adherence: a comprehensive framework for clinical research and practice? A discussion paper. Int J Nurs Stud.

[8] Morisky DE, Green LW, Levine DM (1986). Concurrent and predictive validity of a self-reported measure of medication adherence. Med Care.

[9] Hatah E, Braund R, Tordoff J, Duffull SB (2014). A systematic review and meta-analysis of pharmacist-led fee-for-services medication review. Br J Clin Pharmacol.

[10] Manfrin A, Tinelli M, Thomas T, Krska J (2017). A cluster randomised control trial to evaluate the effectiveness and cost-effectiveness of the Italian medicines use review (I-MUR) for asthma patients. BMC Health Serv Res.

[11] Stewart D, Whittlesea C, Dhital R, Newbould L, McCambridge J. Community pharmacist led medication reviews in the UK: a scoping review of the medicines use review and the new medicine service literatures. Res Social Adm Pharm. 2019. 10.1016/j.sapharm.2019.04.010. 10.1016/j.sapharm.2019.04.010 31085141

[12] Desborough JA, Sach T, Bhattacharya D, Holland RC, Wright DJ (2012). A cost-consequences analysis of an adherence focused pharmacist-led medication review service. Int J Pharm Pract.

[13] Vrijens B, Belmans A, Matthys K, Ed K, Lesaffre E (2006). Effect of intervention through a pharmaceutical care program on patient adherence with prescribed once-daily atorvastatin. Pharmacoepidemiol Drug Saf.

[14] Begley S, Livingstone C, Hodges N, Williamson V (1997). Impact of domiciliary pharmacy visits on medication management in an elderly population. IJPP..

[15] Bouvy ML, Heerdink ER, Urquhart J, Grobbee DE, Hoes AW, Leufkens HG (2003). Effect of a pharmacist-led intervention on diuretic compliance in heart failure patients: a randomized controlled study. J Card Fail.

[16] Elliott RA, Tanajewski L, Gkountouras G, Avery AJ, Barber N, Mehta R (2017). Cost effectiveness of support for people starting a new medication for a long-term condition through community pharmacies: an economic evaluation of the new medicine service (NMS) compared with Normal practice. PharmacoEconomics..

[17] Messerli M, Blozik E, Vriends N, Hersberger KE (2016). Impact of a community pharmacist-led medication review on medicines use in patients on polypharmacy--a prospective randomised controlled trial. BMC Health Serv Res.

[18] Messerli M, Vriends N, Hersberger KE (2018). Humanistic outcomes and patient acceptance of the pharmacist-led medication review "Polymedication check" in primary care in Switzerland: a prospective randomized controlled trial. Patient Prefer Adherence.

[19] Nabergoj Makovec U, Kos M, Pisk N (2018). Community pharmacists' perspectives on implementation of medicines use review in Slovenia. Int J Clin Pharm.

[20] Slovene Chamber of Pharmacies (2016). Medicines Use Review service [in Slovene].

[21] Pisk N, Madjar B, Nabergoj Makovec U, Kos M (2018). Implementation of medicines use review service in Slovenia [in Slovene]. Farm Vest.

[22] Moher D, Hopewell S, Schulz KF, Montori V, Gotzsche PC, Devereaux PJ (2012). CONSORT 2010 explanation and elaboration: updated guidelines for reporting parallel group randomised trials. Int J Surg.

[23] Kim J, Shin W (2014). How to do random allocation (randomization). Clin Orthop Surg.

[24] Pharmacy Practice Act [in Slovene] (2016). Slovenia.

[25] Morisky DE, Ang A, Krousel-Wood M, Ward HJ (2008). Predictive validity of a medication adherence measure in an outpatient setting. J Clin Hypertens (Greenwich).

[26] Krousel-Wood M, Islam T, Webber LS, Re RN, Morisky DE, Muntner P (2009). New medication adherence scale versus pharmacy fill rates in seniors with hypertension. Am J Manag Care.

[27] Morisky DE, DiMatteo MR (2011). Improving the measurement of self-reported medication nonadherence: response to authors. J Clin Epidemiol.

[28] Berlowitz DR, Foy CG, Kazis LE, Bolin LP, Conroy MB, Fitzpatrick P (2017). Effect of intensive blood-pressure treatment on patient-reported outcomes. N Engl J Med.

[29] Bress AP, Bellows BK, King JB, Hess R, Beddhu S, Zhang Z (2017). Cost-effectiveness of intensive versus standard blood-pressure control. N Engl J Med.

[30] Lamina C, Sturm G, Kollerits B, Kronenberg F (2012). Visualizing interaction effects: a proposal for presentation and interpretation. J Clin Epidemiol.

[31] IBM® SPSS® Statistics (2017). 25 ed: IBM Corporate.

[32] Horvat N, Kos M (2016). Development and validation of the Slovenian drug-related problem classification system based on the PCNE classification V 6.2. Int J Clin Pharm.

[33] Katusiime B, Corlett SA, Krska J (2018). Development and validation of a revised instrument to measure burden of long-term medicines use: the living with medicines questionnaire version 3. Patient Relat Outcome Meas.

[34] Nabergoj PP, Makovec U, Kos M, Kerec KM (2018). Living with medicines questionnaire: first insights from Slovenia in 46th ESCP symposium on clinical pharmacy “science meets practice: towards evidence-based clinical pharmacy services”, Heidelberg, Germany, October 9th–11th, 2017. Int J Clin Pharm.

[35] Engels JM, Diehr P (2003). Imputation of missing longitudinal data: a comparison of methods. J Clin Epidemiol.

[36] Hatah E, Tordoff J, Duffull SB, Cameron C, Braund R (2014). Retrospective examination of selected outcomes of medicines use review (MUR) services in New Zealand. Int J Clin Pharm.

[37] Latif A (2017). Community pharmacy medicines use review: current challenges. Integr Pharm Res Pract.

[38] Mehuys E, Van Bortel L, De Bolle L, Van Tongelen I, Annemans L, Remon JP (2008). Effectiveness of pharmacist intervention for asthma control improvement. Eur Respir J.

[39] Hugtenburg JG, Timmers L, Elders PJ, Vervloet M, van Dijk L (2013). Definitions, variants, and causes of nonadherence with medication: a challenge for tailored interventions. Patient Prefer Adherence.

[40] Boeni F, Spinatsch E, Suter K, Hersberger KE, Arnet I (2014). Effect of drug reminder packaging on medication adherence: a systematic review revealing research gaps. Syst Rev.

[41] Rubak S, Sandbaek A, Lauritzen T, Christensen B (2005). Motivational interviewing: a systematic review and meta-analysis. Br J Gen Pract.

[42] Lundahl B, Moleni T, Burke BL, Butters R, Tollefson D, Butler C (2013). Motivational interviewing in medical care settings: a systematic review and meta-analysis of randomized controlled trials. Patient Educ Couns.

[43] Van Wijk BL, Klungel OH, Heerdink ER, de Boer A (2005). Effectiveness of interventions by community pharmacists to improve patient adherence to chronic medication: a systematic review. Ann Pharmacother.

[44] Vrijens B, De Geest S, Hughes DA, Przemyslaw K, Demonceau J, Ruppar T (2012). A new taxonomy for describing and defining adherence to medications. Br J Clin Pharmacol.

[45] Leporini C, De Sarro G, Russo E (2014). Adherence to therapy and adverse drug reactions: is there a link?. Expert Opin Drug Saf.

[46] Kempen TGH, van de Steeg-van Gompel CH, Hoogland P, Liu Y, Bouvy ML (2014). Large scale implementation of clinical medication reviews in Dutch community pharmacies: drug-related problems and interventions. Int J Clin Pharm.

[47] Hammerlein A, Griese N, Schulz M (2007). Survey of drug-related problems identified by community pharmacies. Ann Pharmacother.

[48] Krska J, Avery AJ (2008). Community pharmacy medicines management project evaluation T. evaluation of medication reviews conducted by community pharmacists: a quantitative analysis of documented issues and recommendations. Br J Clin Pharmacol.

[49] Vinks TH, Egberts TC, de Lange TM, de Koning FH (2009). Pharmacist-based medication review reduces potential drug-related problems in the elderly: the SMOG controlled trial. Drugs Aging.

[50] Horvat N, Kos M (2015). Contribution of Slovenian community pharmacist counseling to patients' knowledge about their prescription medicines: a cross-sectional study. Croat Med J.

[51] Puspitasari HP, Aslani P, Krass I (2010). Pharmacists' and consumers' viewpoints on counselling on prescription medicines in Australian community pharmacies. Int J Pharm Pract..

[52] Cline CM, Bjorck-Linne AK, Israelsson BY, Willenheimer RB, Erhardt LR (1999). Non-compliance and knowledge of prescribed medication in elderly patients with heart failure. Eur J Heart Fail.

[53] Nabergoj Makovec U, Janezic A, Kos M (2018). Engaging students in patient studies an experience from randomised controlled trial on medicines use review benefits in Slovenia in abstracts 6th working symposium of the pharmaceutical care network Europe (PCNE), 2–3 February 2018, Fuengirola. Int J Clin Pharmacy.

[54] Nabergoj Makovec U, Horvat N, Kos M (2018). Experiences of the service providers with running a randomised controlled trial on medicines use review service in Slovenia in abstracts 6th working symposium of the pharmaceutical care network Europe (PCNE), 2–3 February 2018. Fuengirola. Int J Clin Pharmacy.

[55] Pednekar PP, Ágh T, Malmenäs M, Raval AD, Bennett BM, Borah BJ (2019). Methods for measuring multiple medication adherence: a systematic review-report of the ISPOR medication adherence and persistence special interest group. Value Health.

[56] Huiskes VJ, Burger DM, van den Ende CH, van den Bemt BJ (2017). Effectiveness of medication review: a systematic review and meta-analysis of randomized controlled trials. BMC Fam Pract.

[57] McCambridge J, Witton J, Elbourne DR (2014). Systematic review of the Hawthorne effect: new concepts are needed to study research participation effects. J Clin Epidemiol.

[58] Mes MA, Katzer CB, Chan AHY, Wileman V, Taylor SJC, Horne R (2018). Pharmacists and medication adherence in asthma: a systematic review and meta-analysis. Eur Respir J.

[59] Janezic A, Locatelli I, Kos M (2017). Criterion validity of 8-item Morisky medication adherence scale in patients with asthma. PLoS One.

[60] Zerovnik S, Kos M (2019). Effective and rationale medicines use review in Slovenia: evidence based policy in abstracts 11th PCNE working conference ‘targeting patients and tailoring pharmaceutical care’. 6–9 February 2019, Egmond aan zee, the Netherlands. Int J Clin Pharm.

