# Supplementary material for: Improved adherence with Medicines Use Review service in Slovenia: a randomized controlled trial
Source: BMC Health Serv Res. 2021 Mar 22;21:266. doi: 10.1186/s12913-021-06223-8 (PMC7986462; doi:10.1186/s12913-021-06223-8)
Supplement: Supplementary file 1 — Additional file 1. Appendix A: Inclusion criteria - examples of patients suitable for the MUR service as per SOP MUR. Appendix B: Handling of missing data. Appendix C: Multiple linear regression models to predict baseline adherence for total ©MMAS-8 score at V1 (N = 129). Appendix D: Baseline population characteristics presented per group and per baseline adherence level (low versus moderate and high) for all patients included in the analysis (N = 140). Appendix E. The detailed results of the secondary outcomes: nature of identified manifested DRPs, risk factors and interventions, ©LMQ and VAS scores and patient information (‘knowledge’) regarding their medicines. [file 12913_2021_6223_MOESM1_ESM.docx]

# APPENDICES

## Appendix A: Inclusion criteria - examples of patients suitable for the MUR service as per SOP MUR

- Patients with polypharmacotherapy (5 or more medicines for regular use)
- Patients with complex dosing regimen or having to take more than 12 doses per day
- Patients use medicines with complex way of administration (inhalers, insulin pens, eye drops, etc.)
- Patients show signs of adherence issues
- Patients show signs or symptoms pointing to potential adverse drug events
- Patients with high-risk medicines for adverse events (use different medicines list like Beers, Priscus criteria, etc.)
- Patients who had bigger changes in therapy in the last three months or more than 4 changes in the last 12 months
- Patients, whose medications are prescribed by several prescribers
- Patients after recent hospital discharge
- Patients with frequent hospitalizations
- Elderly patients (over 65 years)
- Patients, who use several OTCs or other products (food supplements,) for self-medication

## Appendix B: Handling of missing data.

*Eligibility screening:*

Eighteen patients were recorded as being ineligible for the study, although the total number of patients assessed for eligibility was not recorded.

*Characteristics of the drop out patients.*

Thirteen study patients (7 females and 6 males), whose average age was 73.38 (SD= 7.69) years, did not attend V2. Twelve were retired and one choose to not reveal their employment status. They took a median of 7 (range: 3-13) medicines for regular use, resulting in a median of 8.5 (4-23) units of medicine per day. Patients generally reported their health status to be very good (1) or medium (10), with 2 patients choosing not to answer questions regarding health status. Reasons for withdrawal are detailed in Figure 1 in the original manuscript.

*Data imputation to ©MMAS-8 and ©LMQ*

Single answers were missing from ©MMAS-8 questionnaire data from 10 (7%) and 7 (5%) study patients at V1 and V2, respectively.

©LMQ data was imputed for 25 (16%) study patients at V1 and 14 patients (10%) at V2.

## Appendix C: Multiple linear regression models to predict baseline adherence for total ©MMAS-8 score at V1 (N=129)

|  | **Total ©MMAS-8 score at V1** | | |
| --- | --- | --- | --- |
|  | *B* | *95 % CI for B* | *p value* |
| *Constant* | 6.291 | 4.31 - 8.27 | 0.000 |
| *Gender (male vs. female)* | -0.142 | -0.56 - 0.28 | 0.504 |
| *Age^a^ [years]* | -0.004 | -0.03 - 0.02 | 0.761 |
| ***Education****^a^* *[1-4]* | 0.246 | 0.02 - 0.47 | **0.032** |
| *Employment status (retired vs. non-retired)* | 0.393 | -0.33 - 1.12 | 0.286 |
| ***Self-reported current health status^a^*** *[1-5]* | 0.346 | 0.01 - 0.68 | **0.044** |
| *How many times per day patients take their medicines^a^ [1-4]* | -0.298 | -0.65 - 0.06 | 0.102 |
| *Number of Rx medicines for regular use^a^* | 0.076 | -0.01 - 0.17 | 0.093 |
| *Different pharmaceutical forms^b^* | 0.406 | -0.00 - 0.81 | 0.051 |
| *Number of months since the last visit at the GP^a^* | 0.018 | -0.03 - 0.07 | 0.502 |
| *Number of visits in the pharmacy per 3 months^a^* | -0.040 | -0.08 - 0.00 | 0.062 |
| *Model summary* | N=129; R^2^= 0.160; p = 0.020 | | |
| *a - included as scale variable* | | | |
| *b - different pharmaceutical forms vs. only tablets and capsules* | | | |
|  | | | |
| 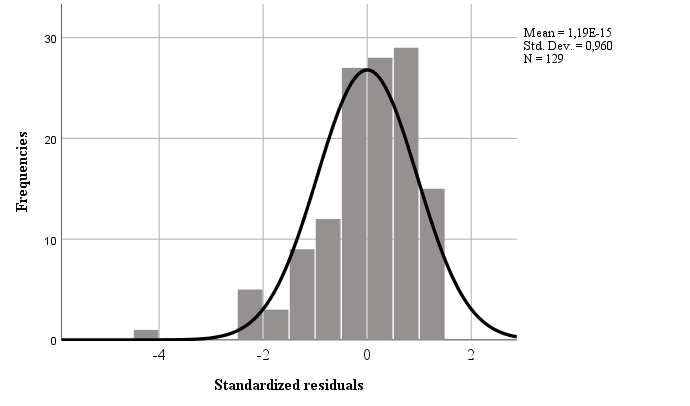 | | | |

*Appendix C - Figure 1. The histogram of standardized residuals of total ©MMAS-8 score at V1*


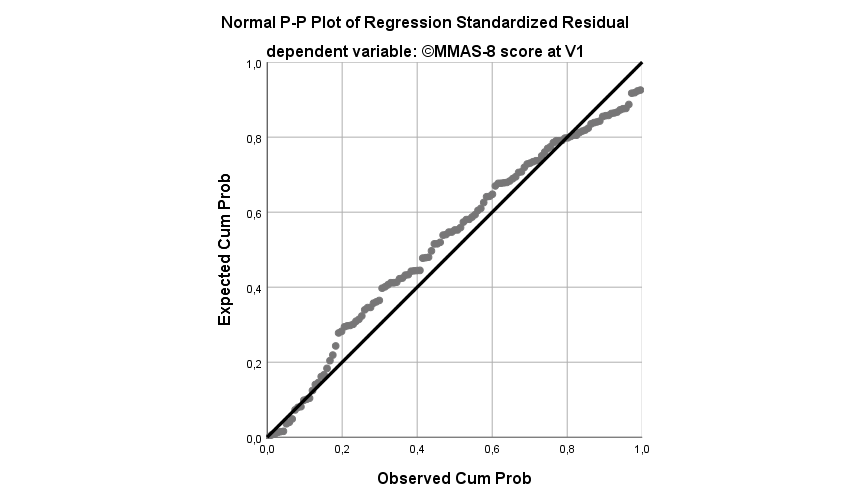
*Appendix C - Figure 2. The normal P-P plot of regression standardized residual of total ©MMAS-8 score at V1.*


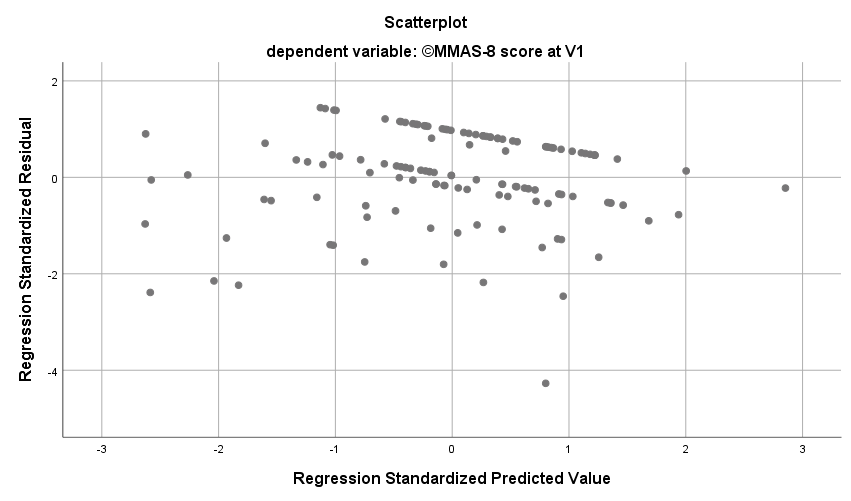
*Appendix C - Figure 3. The scatter plot comparing regression standardized residual to predicted regression standardized residual of total ©MMAS-8 score at V1.*

## Appendix D: Baseline population characteristics presented per group and per baseline adherence level (low versus moderate and high) for all patients included in the analysis (N=140).

| **Baseline characteristics** | **Control group (N= 68)** | | | | **Test group (N=72)** | | | |
| --- | --- | --- | --- | --- | --- | --- | --- | --- |
|  | *Low*  *(N=15)* | | *Medium and high (N=53)* | | *Low*  *(N=10)* | | *Medium and high (N=62)* | |
| Age [years; mean (SD) and range] | 63 (12) | 41-83 | 68 (9) | 46-89 | 73 (8) | 56-84 | 68 (10) | 39-86 |
| *Gender [female; n/%]* | 8 | 53.3 | 27 | 50.9 | 5 | 50.0 | 40 | 64.5 |
| Employment status [retired; n/%] | 10 | 66.7 | 45 | 84.9 | 10 | 100.0 | 53 | 85.5 |
| Education [n/%] |  |  |  |  |  |  |  |  |
| *Elementary school* | 2 | 13.3 | 11 | 20.8 | 2 | 20.0 | 10 | 16.1 |
| *High school* | 9 | 60.0 | 23 | 43.4 | 5 | 50.0 | 33 | 53.2 |
| *College* | 2 | 13.3 | 12 | 22.6 | 2 | 20.0 | 10 | 16.1 |
| *University* | 2 | 13.3 | 7 | 13.2 | 1 | 10.0 | 8 | 12.9 |
| Number of Rx medicines for regular use [median/ range] | 6 | 4-10 | 7 | 2-13 | 7 | 4-12 | 7.00 | 2-13 |
| Different pharmaceutical forms [n/%] |  |  |  |  |  |  |  |  |
| *Different pharmaceutical forms* | 6 | 40.0 | 25 | 47.2 | 4 | 40.0 | 33 | 53.2 |
| *Only tablets and capsules* | 9 | 60.0 | 28 | 52.8 | 6 | 60.0 | 29 | 46.8 |
| (Co)paying for medicines [yes; n/%] | 9 | 60.0 | 31 | 58.5 | 6 | 60.0 | 29 | 46.8 |
| Self-reported current health status [n/%] |  |  |  |  |  |  |  |  |
| *Very poor* | 0 | 0.0 | 0 | 0.0 | 2 | 20.0 | 2 | 3.2 |
| *Poor* | 2 | 13.3 | 3 | 5.7 | 2 | 20.0 | 3 | 4.8 |
| *Medium* | 11 | 73.3 | 31 | 58.5 | 6 | 60.0 | 32 | 51.6 |
| *Good* | 1 | 6.7 | 19 | 35.8 | 0 | 0.0 | 23 | 37.1 |
| *Very good* | 1 | 6.7 | 0 | 0.0 | 0 | 0.0 | 2 | 3.2 |

## Appendix E: The detailed results of the secondary outcomes: nature of identified manifested DRPs, risk factors and interventions, ©LMQ and VAS scores and patient information (‘knowledge’) regarding their medicines.

### Drug related problems

| **Nature of the identified manifested DRPs (N=90)** | **N** | **%** |
| --- | --- | --- |
| P1 Treatment effectiveness | **17** | **18,9** |
| *P1.1 No effect of drug treatment/therapy failure.* | *1* | *1.1* |
| *P1.2 Effect of drug treatment not sufficient or too weak.* | *8* | *8.9* |
| *P1.3 Untreated symptoms or indication.* | *8* | *8.9* |
| P2 Adverse drug event | **54** | **60%** |
| *P2.1 Adverse drug event due to allergic reaction.* | *1* | *1.1* |
| *P2.2 Other adverse drug event.* | *53* | *58.9* |
| P3 Treatment costs | **11** | **12,2%** |
| *P3.1 Drug treatment more costly than necessary* | *8* | *8.9* |
| *P3.2 Unnecessary drug-treatment.* | *3* | *3.3* |
| P4 Others | **8** | **8,9%** |
| *P4.1 Patient dissatisfied with therapy despite optimal clinical and economic treatment outcomes.* | *1* | *1.1* |
| *P4.2 Unclear problem/complaint.* | *7* | *7.8* |

Risk factors for adverse events were undetermined or unclear in 63% of cases, while effectiveness and cost issues were mostly due to prescribing (70% and 64%, respectively). Improper medicine use was a risk factor in 16.7% of all mDRP.

Pharmacists performed 99 interventions: 58 were independent (in 36% referrals to a GP), 23 in agreement with the prescriber, 15 recommendations to the prescriber and 3 adverse events were reported to national pharmacovigilance institution.

### Burden related to daily medicines use (©LMQ)

The average ©LMQ scores were 97.75 (SD=20.31) and 92.88 (SD=19.7) points at V1 for the test and control group, respectively. The burden of daily medicine use was non-existent for 15%, minimal for 56%, moderate for 28%, and high for 1% of patients. The average ©LMQ scores decreased to 93.56 (SD=18.55) and 91.16 (SD=21.33) points at V2 for the test and control group, respectively. The average VAS scores were 2.81 (SD=2.34) and 2.66 (SD=2.13) points at V1, and 2.53 (SD=2.40) and 2.68 (SD=2.25) points at V2, for the test and control group, respectively.

### Patient information (‘knowledge’) regarding their medicines

At the first visit (V1; N=1050 patient-medicines), the purpose of medicine use was stated correctly in 90% of patient-medicines, incorrectly in 4% of patient-medicines, and the accuracy was unconfirmed in 6% of patient-medicines. Patients stated their daily dose correctly in 91% of patient-medicines, incorrectly in 8% of patient-medicines, and the accuracy was unconfirmed in 1% of patient-medicines; among the correctly stated daily doses, the medication regimen was also correctly stated in 85% of patient-medicines.

At second visit (V2; N=939 patient-medicines), 5% more patients correctly stated the purpose of their medicines compared to V1 and 5% more patients incorrectly stated the purpose of their medicines compared to V1, indicating that patient understanding of the purpose of their medicines improved and deteriorated in equal proportions. Daily doses were reported more accurately at V2 compared to V1 in 6% of patient-medicines, and less accurately in 4% of patient-medicines.

Patients stated special warnings regarding their medicines in 15% and 11% of patient-medicines at V1 and V2, respectively; the special warnings were mostly related to specific times that the medicines should be taken, or to the need for regular measurement of blood glucose levels or blood pressure. Among the special warnings, adverse event information was stated in 6.5% and 2% of patient-medicines at V1 and V2, respectively.
