# Supplementary material for: Improved adherence with Medicines Use Review service in Slovenia: a randomized controlled trial
Source: BMC Health Serv Res. 2021 Mar 22;21:266. doi: 10.1186/s12913-021-06223-8 (PMC7986462; doi:10.1186/s12913-021-06223-8)
Supplement: Supplementary file 2 — Additional file 2. Questionnaires used in the Medicines use Review Service in Slovenia: a randomized controlled trial (RCT MUR SLO). [file 12913_2021_6223_MOESM2_ESM.docx]

**QUESTIONNAIRES USED IN THE MEDICINES USE REVIEW SERVICE IN SLOVENIA: A RANDOMIZED CONTROLLED TRIAL (RCT MUR SLO)**

Two questionnaires were used to measure primary or other study outcomes

- The 8-item Morisky Medication Adherence Scale as part of ©Morisky Widget MMAS-8 Software
- The Living with Medicines Questionnaire, v3

The MMAS-8 scoring and coding presented in the article was done using the electronic ©Morisky Widget MMAS-8 Software. Use of the © Morisky Widget MMAS-8 software, copyright registration number TX 8-816-517 is protected by U.S. copyright laws. Permission for use of the Morisky Widget MMAS-8 software is required. A license agreement is available from MMAS Research LLC 14725 NE 20th St Bellevue, WA 98007, USA; [strubow@morisky.org](mailto:strubow@morisky.org)

The Living with Medicines Questionnaire is available from the original authors. Permission to use is required.
