# Supplementary material for: Improved adherence with Medicines Use Review service in Slovenia: a randomized controlled trial
Source: BMC Health Serv Res. 2021 Mar 22;21:266. doi: 10.1186/s12913-021-06223-8 (PMC7986462; doi:10.1186/s12913-021-06223-8)
Supplement: Supplementary file 3 — Additional file 3. CONSORT 2010 checklist for RCT MUR SLO. [file 12913_2021_6223_MOESM3_ESM.pdf]

CONSORT 2010 checklist of information to include when reporting a randomised trial*

Section/Topic	Item No	Checklist item	Reported on page No
**Title and abstract**			
	1a	Identification as a randomised trial in the title	Title
	1b	Structured summary of trial design, methods, results, and conclusions (for specific guidance see CONSORT for abstracts)	Abstract; section methods, results and conclusion
**Introduction**			
Background and objectives	2a	Scientific background and explanation of rationale	Background, spec. L18-24
	2b	Specific objectives or hypotheses	Background, spec. L24-27
**Methods**			
Trial design	3a	Description of trial design (such as parallel, factorial) including allocation ratio	Methods: section *Study design and setting* L31; without allocation ratio.
	3b	Important changes to methods after trial commencement (such as eligibility criteria), with reasons	N/A
Participants	4a	Eligibility criteria for participants	Methods: section *Eligibility criteria* L48-58 and Appendix A.
	4b	Settings and locations where the data were collected	Methods: section *Study design and setting* L31-39
Interventions	5	The interventions for each group with sufficient details to allow replication, including how and when they were actually administered	Methods: section *Intervention* L64-75; section *Study design and*

			setting L32-39; section
Outcomes	6a	Completely defined pre-specified primary and secondary outcome measures, including how and when they were assessed	**Methods:** section *Primary outcome measure* L 76-94 Section *Secondary outcome measure* L95-118
	6b	Any changes to trial outcomes after the trial commenced, with reasons	N/A
Sample size	7a	How sample size was determined	**Methods;** section *Sample size* L119-124
	7b	When applicable, explanation of any interim analyses and stopping guidelines	N/A
Randomisation:			
Sequence generation	8a	Method used to generate the random allocation sequence	**Methods:** section *Eligibility criteria* L59-63
	8b	Type of randomisation; details of any restriction (such as blocking and block size)	**Methods:** section *Eligibility criteria* L59-63
Allocation concealment mechanism	9	Mechanism used to implement the random allocation sequence (such as sequentially numbered containers), describing any steps taken to conceal the sequence until interventions were assigned	N/A
Implementation	10	Who generated the random allocation sequence, who enrolled participants, and who assigned participants to interventions	**Methods:** section *Study design and setting* L31-39
Blinding	11a	If done, who was blinded after assignment to interventions (for example, participants, care providers, those assessing outcomes) and how	N/A
	11b	If relevant, description of the similarity of interventions	N/A
Statistical methods	12a	Statistical methods used to compare groups for primary and secondary outcomes	**Methods:** section *Primary outcome measure* L 76-94

			Section *Secondary outcome measure* L95-118
	12b	Methods for additional analyses, such as subgroup analyses and adjusted analyses	**Methods:** section *Primary outcome measure* L 76-94 Section *Secondary outcome measure* L95-118
**Results**			
Participant flow (a diagram is strongly recommended)	13a	For each group, the numbers of participants who were randomly assigned, received intended treatment, and were analysed for the primary outcome	Results; section *Study population characteristics* L139-141 and **Fig 2** – the CONSORT diagram
	13b	For each group, losses and exclusions after randomisation, together with reasons	Results; section *Study population characteristics* L139-141 and **Fig 2** – the CONSORT diagram and **Appendix B**
Recruitment	14a	Dates defining the periods of recruitment and follow-up	Methods: *Study design and setting* L40.43 and **Fig 1** The RCT timeline. Results: L136-137
	14b	Why the trial ended or was stopped	N/A

Baseline data	15	A table showing baseline demographic and clinical characteristics for each group	Results. **Table 1** and **Appendix D** (table)<br>Section *Study population characteristics* L146-153
Numbers analysed	16	For each group, number of participants (denominator) included in each analysis and whether the analysis was by original assigned groups	**Results:** section *Primary outcome: self-reported adherence according to ©MMAS-8* L158-193; **Table 2** and **table 3** and section *Secondary outcomes* 194-215;<br>**Figure 2**
Outcomes and estimation	17a	For each primary and secondary outcome, results for each group, and the estimated effect size and its precision (such as 95% confidence interval)	**Results:** section *Primary outcome: self-reported adherence according to ©MMAS-8* L158-193; **Table 2** and **table 3, Figure 3** and section *Secondary outcomes* 194-215;

			Appendix C, D, E.
	17b	For binary outcomes, presentation of both absolute and relative effect sizes is recommended	Results: section *Secondary outcomes* 194-215; Appendix E
Ancillary analyses	18	Results of any other analyses performed, including subgroup analyses and adjusted analyses, distinguishing pre-specified from exploratory	Results: section *Primary outcome: self-reported adherence according to ©MMAS-8* L158-193; Appendix C
Harms	19	All important harms or unintended effects in each group (for specific guidance see CONSORT for harms)	N/A
**Discussion**			
Limitations	20	Trial limitations, addressing sources of potential bias, imprecision, and, if relevant, multiplicity of analyses	Discussion: section *Strengths and limitations* L 304-313
Generalisability	21	Generalisability (external validity, applicability) of the trial findings	Discussion: section *Study implementation and population* L279-292; section *Strengths and limitations* L 293-303; section *Implications for pharmacy practice* L322-326
Interpretation	22	Interpretation consistent with results, balancing benefits and harms, and considering other relevant evidence	Discussion;

			section *benefits and challenges of MUR service* L224-278 and section *Study implementation and population* L279-292; section *Implications for pharmacy practice* L315-322
**Other information**			
Registration	23	Registration number and name of trial registry	Abstract – trial registration; Declarations – ethical approval and consent to participate L381-383
Protocol	24	Where the full trial protocol can be accessed, if available	Declarations – ethical approval and consent to participate L381-383
Funding	25	Sources of funding and other support (such as supply of drugs), role of funders	Declarations – Funding L365-369

*We strongly recommend reading this statement in conjunction with the CONSORT 2010 Explanation and Elaboration for important clarifications on all the items. If relevant, we also recommend reading CONSORT extensions for cluster randomised trials, non-inferiority and equivalence trials, non-pharmacological treatments, herbal interventions, and pragmatic trials. Additional extensions are forthcoming: for those and for up to date references relevant to this checklist, see www.consort-statement.org.
